# Controllable La Deficiency Engineering within Perovskite Oxides for Enhanced Overall Water Splitting

**DOI:** 10.3390/molecules29061342

**Published:** 2024-03-18

**Authors:** Xiaohu Xu, Kaiwei Guo, Xinyue Yu

**Affiliations:** College of Physics and Information Engineering, Shanxi Normal University, No. 339 Taiyu Road, Xiaodian District, Taiyuan 030031, China; ewe32e3@163.com

**Keywords:** perovskites, deficiency, hydrogen evolution reaction, oxygen evolution reaction, overall water splitting

## Abstract

Recently, perovskite (ABO_3_) nanomaterials have been widely explored as a class of versatile electrocatalysts for oxygen evolution reactions (OER) due to their remarkable compositional flexibility and structural tunability, but their poor electrical conductivity hinders hydrogen evolution reaction (HER) activity and further limits the large-scale application of perovskite oxide in overall water splitting (OWS). In this study, hollow-nanotube-structure La_x_Co_0.4_Fe_0.6_O_3−δ_ (x = 1.0, 0.9, and 0.8) perovskites with superior HER/OER activity were synthesized on nickel-iron alloy foam (denoted La_x_CoFe/NFF) using hydrothermal with a subsequent calcination strategy. Among them, La_0.9_CoFe/NFF not only exhibited extraordinary HER electrocatalytic performance (160.5 mV@10 mA cm^−2^ and 241.0 mV@100 mA cm^−2^) and stability (20 h@10 mA cm^−2^), but also displayed significant OER electrocatalytic activity (234.7 mV@10 mA cm^−2^ and 296.1 mV@100 mA cm^−2^) and durability (20 h@10 mA cm^−2^), outperforming many recently reported HER/OER perovskite catalysts. The increase in oxygen vacancies caused by the introduction of La deficiency leads to the expansion of the lattice, which greatly accelerates the HER/OER process of La_0.9_CoFe/NFF. Additionally, the naturally porous skeleton can prevent catalysts from aggregating as well as delay the corrosion and dissolution of catalysts in the electrolyte under high applied potentials. Furthermore, the assembled two-electrode configuration, utilizing La_0.9_CoFe/NFF (cathode and anode) electrodes, only requires a low cell voltage of 1.573 V at 10 mA cm^−2^ for robust alkaline OWS, accompanied by remarkable durability over 20 h. This work provides inspiration for the design and preparation of high-performance and stable bifunctional perovskite electrocatalysts for OWS.

## 1. Introduction

Due to the rapid consumption of fossil fuels, associated with the consequent environmental problems, intense efforts have been devoted to exploring clean and renewable energy sources as alternatives [1,2]. Hydrogen as a clean renewable energy source is rising as a star since it possesses numerous superiorities, such as a high calorific value, abundant reserves, and carbon–free emissions in energy storage and conversion [3]. Nevertheless, the conventional industrial production of hydrogen suffers from the depletion of fossil fuel sources and the emissions of carbon dioxide gas [4]. Fortunately, electrochemical water splitting has been widely investigated due to its high purity, low energy consumption, and environmental friendliness. It is composed of two half–cell reactions: the hydrogen evolution reaction (HER) in the cathode and the oxygen evolution reaction (OER) in the anode. As is well known, common electrolyzers are generally assembled based on rare precious metals, such as RuO_2_/IrO_2_ for OER and Pt-based materials for HER, while their high cost, low abundance, and poor stability severely impede their commercial application [5,6,7]. Therefore, the development of bifunctional alternatives with low cost, high activity, and long stability is urgently needed.

In the past decade, extensive efforts have been dedicated to exploiting diverse low-cost, reliable, and high-efficient electrocatalysts for overall water splitting, including transition metal phosphides [8,9], chalcogenides [10,11], hydroxides [12,13], oxides [14,15], etc. Among various materials, perovskite oxide with the general formula of ABO_3_, where A represents a rare-earth or alkaline earth element and B indicates a transition metal ion, has emerged as a promising alternative to precious metal-based catalysts due to their compositional flexibility, antioxidative features, and structural tunability [16,17]. Most recently, some strategies, including defect engineering, elemental doping/substituting, and morphology regulation, have been explored to improve intrinsic catalytic activity. Ideally, the cations at the A and B sites in perovskite oxide are equal to 1, and within a certain deviation range, the perovskite lattice structure can still remain stable due to the high structural tolerance [18]. Previous studies have suggested that engineering A–site deficiency may effectively increase the concentration of the O–site vacancy and modify the electronic structure of B–site cations (e_g_ occupancy) based on the electrical neutrality principle, leading to a change in the physicochemical properties of perovskites and ultimately enhancing OER and oxygen reduction reaction (ORR) catalytic activity [19,20,21]. Specifically, the formation of oxygen vacancies is always accompanied by a change in the e_g_ orbital filling of B–site transition metal. The e_g_ orbital is closely related to the strength of surface anion adsorbate binding, which has a significant influence on the adsorption/desorption properties of intermediates. In addition, the degree of O–site vacancy formation is associated with the covalency between the transition metal 3d bands and the oxygen 2p band, with more covalent systems indicating higher O–site vacancy concentrations for enhanced electrocatalytic activities [21]. In addition, the selective and functional substitution of the B–site in ABO_3_ is also an effective strategy to facilitate OER performance for water splitting [22,23,24]. In particular, LaFeO_3_ is reported as one of the most widely used parent oxides in the perovskite family for applications such as sensors and OER electrocatalysts. All Fe atoms in LaFeO_3_ are located at octahedral sites and the substitution of Fe ions play a vital role in changing Fe-O bonding strength for enhancing the electrocatalytic performances [25,26,27]. Although the above practices have effectively improved the catalytic performances of LaFeO_3_ perovskites, their catalytic activities are still limited by relatively poor conductivity, severely uncontrolled agglomeration, and extremely low specific surface area due to the conventional sol–gel synthesis process [28,29,30]. At the same time, their HER activity in alkaline media is rarely studied previously.

Furthermore, three-dimensional (3D) self–supported electrodes with high porosity have drawn much attention due to their porous interconnected networks, which could provide fast charge and mass transfer via abundant electron/ion pathways during the electrochemical process [31]. At the same time, in situ growth electrocatalysts on macroporous metal foam would endow catalysts with high specific area, excellent electrical conductivity, and numerous inner voids, which would be beneficial to electrolyte permeation and bubble release, hence resulting in the improvement of catalytic activity and mechanical stability [32,33,34,35]. Additionally, the naturally porous skeleton can prevent catalysts from aggregating as well as delay the corrosion and dissolution of catalysts in the electrolyte under high applied potentials [36]. Nevertheless, the majority of currently reported perovskite oxide catalysts are unself–supported powder–like structures, which seriously limits their catalytic performance. Thus, it is necessary to develop self–supported perovskite oxides on the conductive substrate with excellent electronic conductivity to enhance their intrinsic catalytic activities towards overall water splitting.

Inspired by the above findings, we propose an effective strategy to significantly enhance the HER and OER electrocatalytic performances of LaFeO_3_ for overall water splitting by simply engineering A–site deficiency and B–site cation substitution. Hollow nanotube-like perovskite oxides La_x_Co_0.4_Fe_0.6_O_3−δ_ (x = 1.0, 0.9, and 0.8, denoted as LaCoFe/NFF, La_0.9_CoFe/NFF, and La_0.8_CoFe/NFF) were prepared in situ vertically grown on nickel-iron alloy foam (NFF) through mild hydrothermal treatment followed by air annealing. To the best of our knowledge, A–site–deficient perovskites have rarely before been reported as efficient HER catalysts in alkaline media. It was found that La_0.9_CoFe/NFF revealed better HER/OER activity, which could be attributed to the abundant surface oxygen vacancies. More significantly, the assembled two-electrode electrolyzer with La_0.9_CoFe/NFF (cathode and anode) exhibited outstanding catalytic performance and good durability towards overall water splitting (1.573 V @10 mA·cm^−2^ for 20 h).

## 2. Results and Discussion

### 2.1. Structural and Surface Morphology Characterization of Catalysts

**Figure 1:**
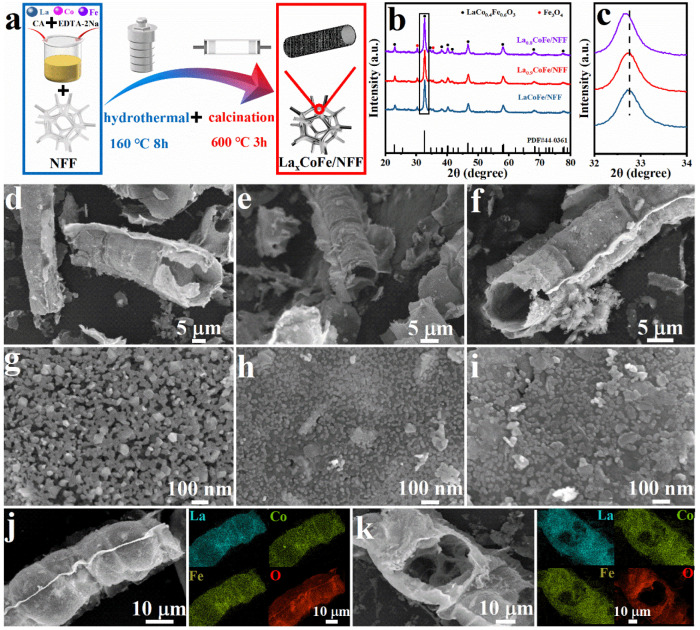
(**a**) Schematic diagram of the synthesis process of the La_x_CoFe/NFF catalyst; (**b**) XRD patterns of the LaCoFe/NFF, La_0.9_CoFe/NFF, and La_0.8_CoFe/NFF; (**c**) enlargement of La_x_CoFe/NFF; SEM images of products obtained at different stages: (**d**,**g**) LaCoFe/NFF, (**e**,**h**) La_0.9_CoFe/NFF, and (**f**,**i**) La_0.8_CoFe/NFF; elemental mapping of La, Co, Fe, and O for (**j**) LaCoFe/NFF and (**k**) La_0.9_CoFe/NFF.

The new La_x_CoFe/NFF catalyst was obtained according to the synthetic route shown in Figure 1a. Perovskite catalysts obtained by conventional synthesis methods have quite low intrinsic electronic conductivities and small specific surface areas, which lead to low catalytic activity, thus limiting their usage. Therefore, in order to improve the performance of the perovskite materials in water splitting, the hydrothermal synthesis method was employed to prepare perovskite catalysts. First, the precursor was synthesized by hydrothermal reaction at 160 °C for 8 h. Subsequently, the hollow nanotube–structured La_x_CoFe/NFF catalyst was obtained by calcination at 600 °C for 3 h in an air atmosphere. The crystal structures of the resultant La_x_CoFe/NFF were characterized by X-ray diffraction (XRD). To eliminate the influence of substrate diffraction peaks, the samples were scraped from the substrate by ultrasound treatment. As shown in Figure 1b, the crystal structures of La_x_Co_0.4_Fe_0.6_O_3−δ_ were investigated by XRD and demonstrated that except for diffraction peaks at 30.3° and 35.4°, which correspond to Fe_3_O_4_ impurities, all other diffraction peaks agree well with LaCoFe standard diffraction peaks (PDF#44-0361, denoted by the ICDD, the International Centre of Diffraction Data). These diffraction peaks reveal an orthorhombic perovskite structure with a space group of Pnma. This reveals that the orthorhombic structure of perovskites can be stabilized even when introducing an A–site deficiency. Additionally, as shown in the enlarged XRD pattern from 32° to 34° (Figure 1c), the main diffraction peaks of the La_x_CoFe catalyst after the introduction of A–site deficiency shifted to a lower angle, which indicates the lattice expansion of catalysts. Furthermore, the particle sizes of the catalysts were obtained by the Scherrer formula based on the main peak of the (112) plane, while the corresponding crystallite sizes of LaCoFe/NFF, La_0.9_CoFe/NFF, and La_0.8_CoFe/NFF were 12.75, 13.36, and 13.78 nm, respectively [23]. This phenomenon can be attributed to the effect of A–site deficiency, where the average grain size shows a positive correlation with changes in A–site content.

**Figure 2:**
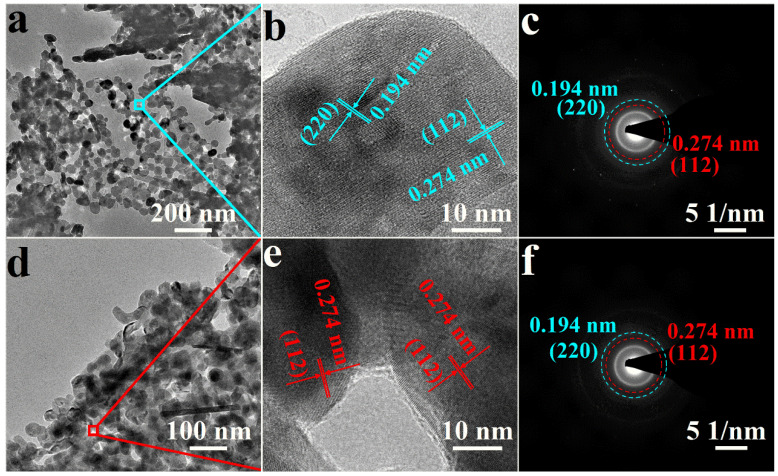
TEM images of (**a**) LaCoFe, (**d**) La_0.9_CoFe; HRTEM of (**b**) LaCoFe, (**e**) La_0.9_CoFe; and SAED of (**c**) LaCoFe, (**f**) La_0.9_CoFe.

The scanning electron microscope (SEM) and transmission electron microscope (TEM) images of the as–synthesized La_x_CoFe/NFF hollow nanotube are presented in Figure 1d–k and Figure 2. Figure 1d shows the SEM image of the broken perovskite nanotube, which clearly shows the hollow structure. Moreover, Figure 1g displays a high–magnification image of the hollow nanotube surface, revealing numerous uniformly distributed and smooth surface nanoparticles (with diameters ranging between 20 and 50 nm). The structures of La_0.9_CoFe/NFF and La_0.8_CoFe/NFF, maintained as hollow nanotubes, can be observed to evolve with the increasing A–site defects, as shown in Figure 1e,h,f,i. The hollow structure of the nanotubes contributes to a large specific surface area, which is advantageous to promote electron transport through the abundant active sites and then improve the catalytic performance. Nanotubes with a hollow structure can provide a large specific surface area. In addition, the EDS spectra of LaCoFe/NFF and La_0.9_CoFe/NFF shown in Figure 1j,k indicate that all elements are evenly well distributed.

To further characterize the morphology and crystal structure of La_x_CoFe, TEM measurement was also adopted. As shown in Figure 2a,d, it can be observed that small particles agglomerated together after high–temperature sintering. Further, HRTEM studies were carried out on LaCoFe and La_0.9_CoFe to understand the nature of the particles. Specifically, as shown in Figure 2b,e, the lattice fringe analysis of the sample displays the interplane spacing of 0.274 and 0.194 nm, corresponding to (112) and (220) of LaCoFe, which further confirms the XRD result, indicating that LaCo_0.4_Fe_0.6_O_3_ was successfully synthesized. The obvious set of diffraction rings can be revealed from selected area electron diffraction, being assigned to the (112) and (220) planes of LaCoFe, confirming the polycrystalline nature of the LaCoFe nanoparticles (Figure 2c,f).

### 2.2. Characterization of Surface Valence States of Catalysts

**Figure 3:**
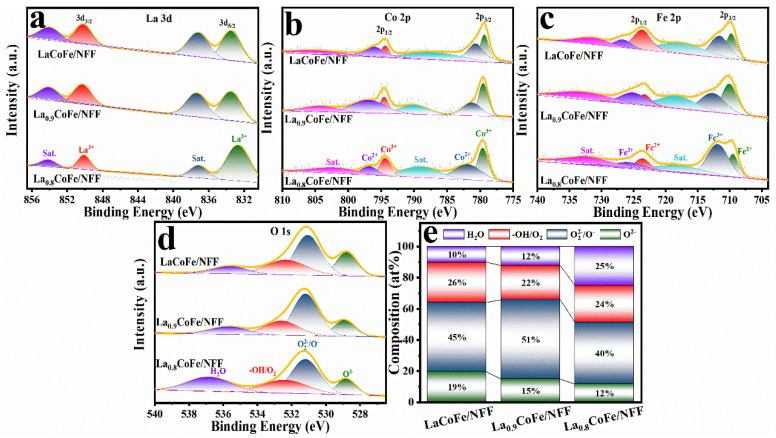
XPS spectra of (**a**) La 3d; (**b**) Co 2p; (**c**) Fe 2p; (**d**) O 1s spectrum from LaCoFe/NFF, La_0.9_CoFe/NFF, and La_0.8_CoFe/NFF, respectively, and (**e**) surface O atomic composition from XPS of LaCoFe/NFF, La_0.9_CoFe/NFF, and La_0.8_CoFe/NFF.

After gaining significant insights into the structural and morphological aspects of the catalyst, the surface chemical states and compositions of the as–prepared samples were further identified by X-ray photoelectron spectroscopy (XPS). The XPS survey spectra in Figure S1 suggest the coexistence of La, Co, Fe, and O elements in La_x_CoFe/NFF, which coincides well with the EDS element mapping analysis. Specifically, in the La 3d spectra (Figure 3a), each spectrum is illustrated by two doublet peaks in the 832–840 eV and 848–856 eV regions, corresponding to the La 3d_5/2_ and La 3d_3/2_ orbitals, respectively. Within the two doublet peak regions, the main peak located at the lower binding energy side is attributed to La^3+^, and the peak at the higher binding energy site belongs to their satellite peaks. The difference in the binding energy (∆) between the La 3d_5/2_ and La 3d_3/2_ main peaks corresponds to the spin–orbit coupling of La. Further, the ∆ value of La in all three materials is in the range of 16.5–17.5 eV. The positions and ∆ values of the La 3d_5/2_ and La 3d_3/2_ binding energy peaks are significantly distinctive of La in the +3 oxidation state [37,38,39].

Figure 3b illustrates the Co 2p XPS spectrum. Two peaks were observed at 780 eV and 795 eV, which correspond to Co 2p_3/2_ and Co 2p_1/2_, respectively. Both the Co 2p_3/2_ and Co 2p_1/2_ orbitals could be deconvoluted into Co^3+^, Co^2+^, and satellite peaks. The Co^2+^ and Co^3+^ ratios of those catalysts can be estimated through the relative peak areas, which correspond to Co^2+^/Co^3+^ results of 1.44 for LaCoFe/NFF, 1.22 for La_0.9_CoFe/NFF, and 1.71 for La_0.8_CoFe/NFF [23]. The average valence states of Co in LaCoFe/NFF, La_0.9_CoFe/NFF, and La_0.8_CoFe/NFF were 2.41, 2.45, and 2.36, respectively, which were obtained from the respective ratios. At the same time, under the assumption that Co^3+^ (e_g_ = 1) goes out to the low spin state and Co^2+^ (e_g_ = 2) is in the high spin state, the e_g_ occupancies of LaCoFe/NFF, La_0.9_CoFe/NFF, and La_0.8_CoFe/NFF can be obtained as 1.59, 1.54, and 1.63, respectively. Thus, the e_g_ of La_0.9_CoFe/NFF is the closest to 1.2 (perovskites with the optimal e_g_ ≈ 1.2 show landmark intrinsic OER activity compared with IrO_2_ according to previous studies) [23,25]. The XPS spectra of Fe 2p were also presented. In Figure 3c, the Fe 2p_3/2_ and Fe 2p_1/2_ peaks could be presented for all of the La_x_CoFe/NFF compounds due to spin-orbit doublet splitting [40,41]. In the spectra, both the Fe 2p_3/2_ and Fe 2p_1/2_ peaks could be deconvoluted into two separate peaks of Fe^3+^ and Fe^2+^, with a higher energy of Fe^3+^ compared to Fe^2+^. Additionally, a pair of satellite peaks exist in the range of 717.6–718.2 eV and 731.4–732.6 eV [42]. As shown in Figure 3d, the O 1s spectra of all the La_x_CoFe/NFF perovskite catalysts can be resolved into four peaks corresponding to lattice oxygen species (O^2−^), highly oxidative oxygen species ($O_{2}^{2-}$/O_2_), hydroxyl groups or oxygen (−OH/O_2_), and adsorbed molecular water (H_2_O), respectively [43]. In addition, oxygen vacancies can be easily introduced into the material, which helps to modify the B–O bond and the surface configuration, promoting the performance of OER. All related peak positions are also listed in Supplementary Materials: Table S1.

Figure 3e summarizes the surface atomic composition of these different oxygen-related species. Previous studies suggested that the relative content of $O_{2}^{2-}$/O_2_ species is associated with surface oxygen vacancies. The content of $O_{2}^{2-}$/O_2_ is calculated to be 51% for the La_0.9_CoFe/NFF catalysts, which is higher than that of the LaCoFe/NFF (45%) and La_0.8_CoFe/NFF (40%) [44]. This demonstrates that the introduction of La deficiency could contribute to generating more oxygen vacancies in the catalysts, which is beneficial to the enhancement of HER/OER activity [45]. Based on the above–mentioned analyses, it can be concluded that more rich oxygen vacancies can be generated in the La_0.9_CoFe/NFF catalysts. These vacancies serve as the active sites for perovskite–based catalysts in the processes of HER/OER, thereby contributing to enhanced catalytic activities [43].

### 2.3. Electrocatalytic Performance for HER in Alkaline

**Figure 4:**
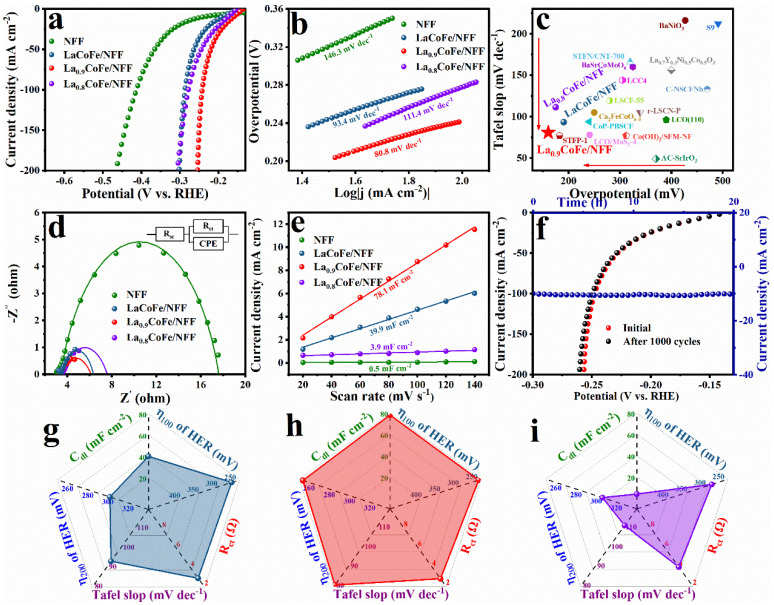
HER performance of the as–prepared catalysts for (**a**) polarization curves; (**b**) Tafel plots of the corresponding samples; (**c**) comparisons of the HER performances with regard to kinetics and at an overpotential of 10 mA cm^−2^ for LaCoFe/NFF, La_0.9_CoFe/NFF, La_0.8_CoFe/NFF with perovskite electrocatalysts reported previously; (**d**) nyquist plots of the corresponding samples; (**e**) C_dl_ of the corresponding samples; (**f**) HER polarization curves of La_0.9_CoFe/NFF before and after 1000 cyclic voltammetry scanning, and the chronoamperometric curve at overpotential of 160.5 mV and comparison of C_dl_, overpotential (η_100_ and η_200_), R_ct_, and Tafel slope of (**g**) LaCoFe/NFF, (**h**) La_0.9_CoFe/NFF, (**i**) La_0.8_CoFe/NFF.

The electrocatalytic activities of HER and OER were evaluated using La_x_CoFe/NFF directly as working electrodes in a 1 M KOH electrolyte with iR correction. The carbon rod and Hg/HgO were used as the counter and reference electrodes, respectively. Firstly, the LSV polarization curves of LaCoFe/NFF for HER/OER under different hydrothermal temperatures and ratios of Co/Fe were studied. The sample exhibited the best HER/OER performance at 160 °C (Figure S2), and the optimal molar ratio of Co/Fe is 4:6 (Figure S3). Additionally, the LSV curves of LaCoFe synthetized on NF (denoted as LaCoFe/NF) with the same production are compared with LaCoFe/NFF, as shown in Figure S4. LaCoFe/NFF displayed better HER/OER performance than LaCoFe/NF. The HER performance of NFF and La_x_CoFe/NFF was displayed in Figure 4a. Specifically, an overpotential of 160.5 mV is achieved for La_0.9_CoFe/NFF at 10 mA cm^−2^ current density, which is superior to the 190.5 mV for LaCoFe/NFF, 174.9 mV for La_0.8_CoFe/NFF, and 240.1 mV for NFF, indicating that La_0.9_CoFe/NFF displays the optimal HER activity among the La_x_CoFe/NFF samples. The HER electrocatalytic kinetics of the developed electrocatalysts were further evaluated using Tafel plots. Obviously, La_0.9_CoFe/NFF possessed the smallest Tafel slope of 80.8 mV dec^−1^, which was apparently lower than that of LaCoFe/NFF (93.4 mV dec^−1^), La_0.8_CoFe/NFF (111.4 mV dec^−1^), and NFF (146.3 mV dec^−1^), suggesting that La_0.9_CoFe/NFF had the faster reaction rate and kinetics (Figure 4b). In respect of the low overpotential and Tafel slope, La_0.9_CoFe/NFF is superior to most reported perovskite oxide–based HER electrocatalysts, as depicted in Figure 4c and Table S2. EIS was tested to further understand the charge-transfer kinetics of the catalysts. Figure 4d reveals that the R_ct_ value of La_0.9_CoFe/NFF (2.81 Ω) is much smaller than those of LaCoFe/NFF (2.93 Ω), La_0.8_CoFe/NFF (4.02 Ω) and NFF (14.21 Ω), proving faster charge transfer and more facile kinetics during HER.

To further evaluate HER activity, the electrochemical double–layer capacitance (C_dl_) was estimated by the cyclic voltammogram (CV) curves of the as–fabricated electrocatalysts (Figure S5). As depicted in Figure 4e, the C_dl_ values of La_0.9_CoFe/NFF were 78.1 mF·cm^−2^, which was higher than those of LaCoFe/NFF (39.9 mF·cm^−2^), La_0.8_CoFe/NFF (3.9 mF·cm^−2^), and NFF (0.5 mF·cm^−2^). This indicates that the La_0.9_CoFe/NFF hollow nanotubes possess the maximum electrochemically active surface area (ECSA) (Figure S6). Furthermore, to obtain a certain idea of the intrinsic catalytic activity, the turnover frequency (TOF) was further calculated. As shown in Figures S7 and S8, La_0.9_CoFe/NFF exhibits a significant TOF value of 0.64 s^−1^ (η = 200 mV), which is 3.8 and 4.6 times higher than those of LaCoFe/NFF (0.17 s^−1^) and La_0.8_CoFe/NFF (0.14 s^−1^), respectively, indicating the outstanding intrinsic activity of La_0.9_CoFe/NFF. In addition, the LSV curves of La_0.9_CoFe/NFF measured before and after 1000 CV cycling tests almost overlap, which corroborates the remarkable long–term HER stability. At the same time, La_0.9_CoFe/NFF displays negligible attenuation using the chronoamperometry test under a constant current density of 10 mA·cm^−2^ for 20 h (Figure 4f). Additionally, Figure S9 depicts the chronopotentiometry tests performed for potential at a current density of −10 mA cm^−2^ for 20 h in 1 M KOH electrolyte, further suggesting its excellent HER activity and exceptional durability. The radar charts show the comparison for the overpotential (η_100_ and η_200_), Tafel slop, C_dl_ and R_ct_ as presented in Figure 4g–i, where the larger area of the closed loop for La_0.9_CoFe/NFF means better comprehensive catalytic performance. In addition, the specific surface area of the samples was analyzed based on the N_2_ adsorption–desorption experiment by the Brunauer–Emmett–Teller (BET) method. As expected, the BET surface area of La_0.9_CoFe/NFF (8.3 m^2^/g) is found to be higher than that of LaCoFe/NFF (6.3 m^2^/g) and La_0.8_CoFe/NFF (6.6 m^2^/g) (Figure S10a–c). Likewise, Figure S10d displayed the specific activity (SA, normalized by real oxide surface area obtained from BET results) of LaCoFe/NFF, La_0.9_CoFe/NFF, and La_0.8_CoFe/NFF at an overpotential of 200 mV. Distinctly, La_0.9_CoFe/NFF still possessed the highest value among all samples. Based on the above characterization results, La_0.9_CoFe/NFF hollow nanotubes exhibited outstanding HER performance, which is mainly attributed to the enhanced oxygen vacancy, faster charge transfer, and higher ECSA. Moreover, the morphology and composition of the La_0.9_CoFe/NFF after the stability test were observed through SEM and XPS (Figures S11 and S12). They remained insignificant changes after HER stability, further demonstrating the robust operational stability of La_0.9_CoFe/NFF.

### 2.4. Electrocatalytic Performance for OER and OWS in Alkaline

**Figure 5:**
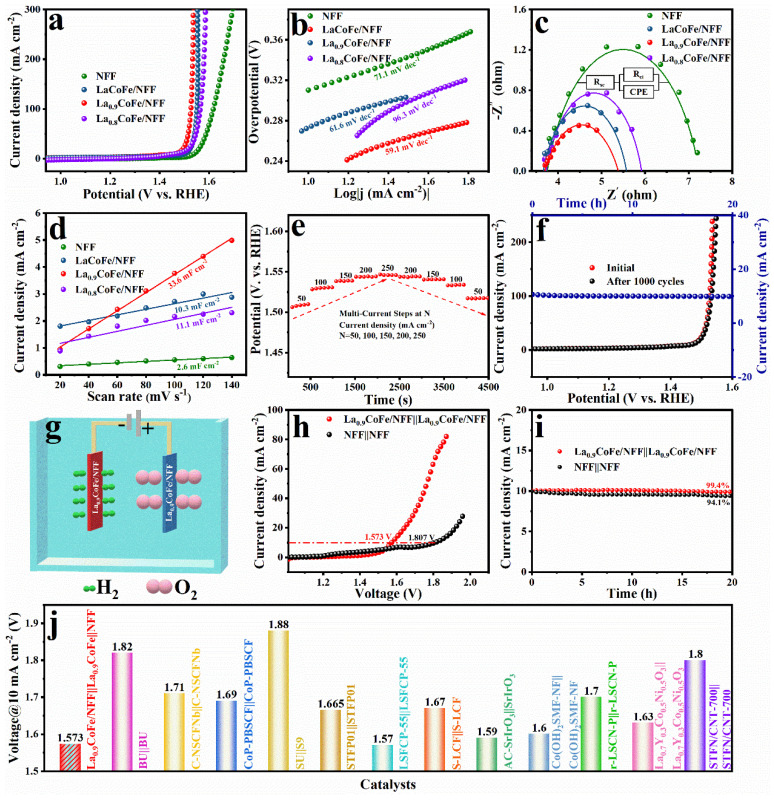
OER performance of the as-prepared catalysts for (**a**) polarization curves; (**b**) Tafel plots; (**c**) nyquist plots of the corresponding samples; (**d**) C_dl_ value of the corresponding catalysts at a specific potential; (**e**) the multi-current process of La_0.9_CoFe/NFF (50–250 mA cm^−2^); (**f**) OER polarization curves of La_0.9_CoFe/NFF before and after 1000 cyclic voltammetry scanning, and the chronoamperometric curve at overpotential of 234.7 mV; (**g**) schematic diagram of the overall water electrolysis cell; (**h**) overall water splitting performance of La_0.9_CoFe/NFF||La_0.9_CoFe/NFF electrolyzer and NFF||NFF electrolyzer; (**i**) chronoamperometric test of overall electrochemical water splitting on La_0.9_CoFe/NFF||La_0.9_CoFe/NFF electrolyzer (at 1.573 V) and NFF||NFF electrolyzer (at 1.807 V) and (**j**) comparison of La_0.9_CoFe/NFF||La_0.9_CoFe/NFF with other bifunctional perovskite electrocatalysts.

Analogously, the OER catalytic performance of the as–fabricated electrocatalysts in a 1 M KOH solution was also measured. The LSV curves show that La_0.9_CoFe/NFF requires an overpotential of 296.1 mV to reach 100 mA cm^−2^, lower than LaCoFe/NFF (321.4 mV), La_0.8_CoFe/NFF (334 mV), and NFF (387.5 mV) (Figure 5a). The Tafel curves of LaCoFe/NFF, La_0.9_CoFe/NFF, and La_0.8_CoFe/NFF and NFF are shown in Figure 5b. The Tafel slope of La_0.9_CoFe/NFF (59.1 mV dec^−1^) considerably outperforms those of LaCoFe/NFF (61.6 mV dec^−1^), La_0.8_CoFe/NFF (96.3 mV dec^−1^) and NFF (71.1 mV dec^−1^), revealing the significantly accelerated catalytic reaction kinetics of La_0.9_CoFe/NFF. In Figure 5c, La_0.9_CoFe/NFF possesses the lowest R_ct_ of about 1.69 Ω among the control catalysts (LaCoFe/NFF: 1.91 Ω, La_0.8_CoFe/NFF: 2.19 Ω, NFF: 3.48 Ω). It indicates that La_0.9_CoFe/NFF exhibited a smaller charge transfer resistance and faster charge transport ability. By analyzing the CV curves at different scan rates, the ECSA was estimated using the C_dl_ to evaluate the inherent activity of the electrocatalysts (Figure S13). The La_0.9_CoFe/NFF catalyst exhibits larger C_dl_ and ECSA (33.6 mF·cm^−2^, 840 cm^2^) than those of LaCoFe/NFF (10.3 mF·cm^−2^, 257.5 cm^2^), La_0.8_CoFe/NFF (11.1 mF·cm^−2^, 277.5 cm^2^) and NFF (2.6 mF·cm^−2^, 65 cm^2^), as shown in Figure 5d and Figure S14. It reflects that more active sites exist in La_0.9_CoFe/NFF to promote its OER activity. As shown in Figure S15, the La_0.9_CoFe/NFF electrode has the largest TOF value of 0.88 s^−1^ (η = 300 mV), which shows that there is an approximately 3, 8, and 14 times increment for La_0.9_CoFe/NFF compared to the LaCoFe/NFF, La_0.8_CoFe/NFF, and NFF, respectively, implying better intrinsic activity. Moreover, the La_0.9_CoFe/NFF shows an outstanding SA value of 2.93 mA cm^−2^ (η = 270 mV), which is 1.99 and 1.04 times higher than those of LaCoFe/NFF (1.47 mA cm^−2^) and La_0.8_CoFe/NFF (2.82 mA cm^−2^), respectively, indicating the excellent intrinsic activity of La_0.9_CoFe/NFF for OER (Figure S16). To demonstrate its excellent superiority for OER, the performance of La_0.9_CoFe/NFF is comparable with other perovskite electrocatalysts reported in the literature (Table S3).

Stable durability and high current density tolerance are remarkable characteristics in practical scenarios. Figure 5e displays a multi–current OER process that ranges from 50 mA cm^−2^ to 250 mA cm^−2^ and back to 50 mA cm^−2^, whereas there is no evident voltage decay, reflecting the fact that the great majority of active sites on La_0.9_CoFe/NFF were maintained after a vigorous bubble release process, thus confirming superior tolerance for the high current density of the La_0.9_CoFe/NFF electrode in industrial applications. Figure 5f illustrates that the LSV curve displayed no obvious decay in the OER activity of La_0.9_CoFe/NFF after 1000 CV cycles, and the catalytic activity can be retained with a negligible decrease for at least 20 h of constant overpotentials of 234.7 mV. Similarly, Figure S7 depicts the chronopotentiometry tests performed for potential at a current density of 10 mA cm^−2^ for 20 h in 1 M KOH electrolyte. The SEM images (Figure S18) and XPS spectra (Figure S19) after the OER stability test also indicated almost no change for the catalyst.

Inspired by the remarkable OER and HER activity (La_0.9_CoFe/NFF), a home–made two-electrode system was assembled with La_0.9_CoFe/NFF serving as the cathode and anode, respectively, as schematically represented in Figure 5g. The NFF||NFF electrolyzer achieved a current density of 10 mA cm^−2^ at 1.807 V, while the La_0.9_CoFe/NFF||La_0.9_CoFe/NFF electrolyzer achieved the same at only 1.573 V in Figure 5h. Moreover, the long-term stability test of La_0.9_CoFe/NFF||La_0.9_CoFe/NFF at 10 mA·cm^−2^ for 20 h was also conducted with small potential loss, which is superior to that of NFF||NFF (Figure 5i). This OWS activity also exceeds that of most HER/OER bifunctional perovskite electrocatalysts (Figure 5j and Table S4), suggesting a wide range of industrial application prospects in electrochemical OWS.

## 3. Experimental Section

### 3.1. Materials

Nickel foam (NF) and nickel-iron alloy foam (NFF) were obtained from KunShan Kunag Xun Electronics Co., Ltd (Suzhou, China,). Acetone, hydrochloric acid, anhydrous ethanol, potassium hydroxide, lanthanum nitrate hexahydrate (La(NO_3_)_3_·6H_2_O), cobaltous nitrate hexahydrate (Co(NO_3_)_2_·6H_2_O), iron nitrate nonahydrate (Fe(NO_3_)_3_·9H_2_O), citric acid (CA), and ethylenediaminetetraacetic acid disodium salt dihydrate (EDTA-2Na) were purchased from Sinopharm Chemical Reagent Co., Ltd(Shanghai, China). All chemicals were of analytical grade and directly used without further treatments. Deionized (DI) water with a resistivity of 18.2 MΩ cm^−1^ used in all experiments was purified through a Millipore system.

### 3.2. Preparation of La_x_CoFe/NFF Catalyst

In a typical procedure, NFF (2 × 3 cm) was cleaned by acetone and hydrochloric acid (3.0 M) to remove the surface oxides and impurities, then rinsed with DI water and alcohol. The stoichiometric amounts of La(NO_3_)_3_·6H_2_O, Co(NO_3_)_2_·6H_2_O and Fe(NO_3_)_3_·9H_2_O were dissolved in 30 mL DI water. Then, certain amounts of EDTA–2Na and CA were added to the metal precursor solution, which are used as the complexing agents. The molar ratio of EDTA–2Na, CA, and total metal ions was 1:2.5:1. Later, transfer the above solution to a 50 mL Teflon–lined autoclave with NFF for 8 h at 160 °C to prepare the precursor. The obtained precursor was annealed at 600 °C for 3 h in air with a ramping rate of 2.5 °C min^−1^. At last, the samples were cooled to room temperature in the furnace to obtain the La_x_CoFe/NFF perovskite catalysts, named LaCoFe/NFF, La_0.9_CoFe/NFF, and La_0.8_CoFe/NFF, respectively.

### 3.3. Characterization

The morphology of all samples was investigated by field–emission scanning electron microscopy (FE–SEM, Hitachi, SU–8010, Tokyo, Japan) and high–resolution transmission electron microscopy (HR–TEM, JEM–2100, 200 kV, Tokyo, Japan) with X-ray energy–dispersive spectroscopy. The crystal diffraction patterns of samples were recorded by an X-ray diffractometer (XRD, Bruker D8–Advance, Hsinchu, Taiwan) equipped with a Cu Kα radiation source (*λ* =1.5418 Å). The particle size was calculated by the Scherrer equation, *D* = *Kλ*/(*B* cos *θ*), where B is the full width at half-maximum height of the peaks, K is the spherical shape factor (0.89), and *D* is the linear dimension of the particle. The surface composition and valence state of the samples were characterized by X-ray photoelectron spectroscopy (XPS, Kratos Axis Ultra DLD, Manchester, UK). The nitrogen adsorption–desorption isotherms were carried out by the ASAP 2460 automatic volumetric sorption analyzer(Norcross, GA, USA) at 77 K. The specific surface area and the corresponding pore size distribution of catalysts were investigated by utilizing the Brunauer–Emmett–Teller (BET) method.

### 3.4. Electrochemical Measurements

All electrochemical data tests were performed using an electrochemical workstation (CHI 760E, CH Instruments, Shanghai, China) with a three–electrode cell configuration. The obtained catalysts on NFF (or NF), a mercury oxide electrode (Hg/HgO) and a carbon rod (4 mm in diameter) were directly used as the working electrode, the reference electrode, and the counter electrode, respectively. All electrochemical measurements were conducted under the same conditions in 1.0 M KOH. Before the linear sweep voltammetry (LSV) tests, the working electrodes were activated by using the cyclic voltammetry (CV) test at a scanning rate of 100 mV s^−1^ for a period of time. Then, LSV measurements for OER were conducted at a potential ranging from 0.0 to 1.0 V (vs. Hg/HgO) at a scanning rate of 1 mV s^−1^ in an O_2_ saturated 1.0 M KOH. LSV measurements for HER were conducted at a potential ranging from −1.5 to −1 V (vs. Hg/HgO) at a scanning rate of 1 mV s^−1^ in an O_2_ saturated 1.0 M KOH. Electrochemical impedance spectroscopy (EIS) experiments were performed in the frequency range from 100 KHz to 1 Hz with an amplitude potential of 5 mV. When the signals of the working electrodes stabilized after scanning several times, the data were collected. The stability test was implemented using the chronopotentiometric method (j–t) at certain potentials and multi–current step measurements without adding electrolyte midway. All the potentials with regard to Hg/HgO reported in this work were converted to the reversible hydrogen electrode (RHE) according to the following equation: E (RHE) = E (vs. Hg/HgO) + 0.059 × pH+ 0.098. All of the above measurements were corrected by manual iR compensation using the current and the solution resistance. The electrochemical double-layer capacitance (C_dl_) of catalysts was measured by performing CV in the non-faradaic potential range of 0.57 to 0.67 V vs. RHE (HER) and 1.12 to 1.26 V vs. RHE (OER) at different scanning rates (v) of 20 to 140 mV s^−1^, followed by the extraction of the slope from the resulting |*j*_a_ − *j*_c_|/2 versus v plots, where *j*_a_ and *j*_c_ represent the anodic and cathodic current densities at 0.62 V vs. RHE (HER) and 1.19 V vs. RHE (OER).

## 4. Conclusions

In summary, we have successfully synthesized hollow–nanotube La_x_Co_0.4_Fe_0.6_O_3−δ_ (x = 1.0, 0.9, and 0.8) perovskite on nickel–iron alloy foam substrate (denoted La_x_CoFe/NFF) by hydrothermal process and subsequent calcination strategy. With the introduction of La deficiency into LaCoFe/NFF, the optimal La_0.9_CoFe/NFF electrode displays excellent bifunctional electrocatalytic activities towards HER and OER, along with robust long–term stability. The increase in oxygen vacancies caused by the introduction of La deficiency greatly accelerated the HER/OER process of La_0.9_CoFe/NFF. Furthermore, a two–electrode cell employing La_0.9_CoFe/NFF as both anode and cathode for OWS was also constructed, where only an ultralow cell voltage of 1.573 V is required to drive the current density of 10 mA·cm^−2^, accompanied by excellent durability. This work opens a new avenue for designing low–cost perovskite oxides with hollow nanotubes and developing highly active bifunctional electrocatalysts based on the perovskite transition metal oxides for both OER and HER applications.

## Data Availability

Data are contained within the article and Supplementary Materials.

## Supplementary Materials

The following supporting information can be downloaded at: https://www.mdpi.com/article/10.3390/molecules29061342/s1, Figure S1: Overall XPS survey spectra of LaCoFe/NFF, La0.9CoFe/NFF and La0.8CoFe/NFF; Figure S2: LSV curves of LaCoFe/NFF catalysts with different hydrothermal temperature in 1 M KOH solution for (a) HER and (b) OER; Figure S3: LSV curves of various catalysts with different Co/Fe ratio in 1 M KOH solution for (a) HER and (b) OER; Figure S4: LSV curves of LaCoFe/NF and LaCoFe/NFF in 1 M KOH solution for (a) HER and (b) OER; Figure S5: Cyclic voltammetry (CVs) curves for HER in a non-faradic current region (potential window at 0.57–0.67 V (vs. RHE)) at different scan rates (20, 40, 60, 80, 100, 120 and 140 mV s^−1^) of (a) NFF, (b) LaCoFe/NFF, (c) La0.9CoFe/NFF and (d) La0.8CoFe/NFF; Figure S6: Electrochemical active area (ECSA) of HER; Figure S7: Reduction peaks recorded at 0.2 V s^−1^. (a) NFF; (b) LaCoFe/NFF; (c) La0.9CoFe/NFF; (d) La0.8CoFe/NFF; Figure S8: (a) The potential dependent TOF curves of the NFF, LaCoFe/NFF, La0.9CoFe/NFF and La0.8CoFe/NFF; (b) TOF values at the overpotential of 200 mV of the corresponding samples; Figure S9: Chronopotentiometry test for 20 h at −10 mA cm^−2^ of electrocatalysts; Figure S10: BET surface area calculations from the N2 absorption-desorption isotherms. (a) LaCoFe/NFF, (b) La0.9CoFe/NFF and (c) La0.8CoFe/NFF; (d) Specific activity of LaCoFe/NFF, La0.9CoFe/NFF and La0.8CoFe/NFF recorded at an overpotential of 200 mV; Figure S11: SEM images of La0.9CoFe/NFF after HER long-term tests in 1 M KOH; Figure S12: XPS spectra of (a) La 3d, (b) Co 2p (c) Fe 2p and (d) O 1s of La0.9CoFe/NFF after HER tests in 1M KOH; Figure S13: Cyclic voltammetry (CVs) curves for OER in a non-faradic current region (potential window at 1.12–1.26 V (vs. RHE)) at different scan rates (20, 40, 60, 80, 100, 120 and 140 mV s^−1^) of (a) NFF, (b) LaCoFe/NFF, (c) La0.9CoFe/NFF and (d) La0.8CoFe/NFF; Figure S14: Electrochemical active area (ECSA) of OER; Figure S15: (a) The potential dependent TOF curves of the NFF, LaCoFe/NFF, La0.9CoFe/NFF and La0.8CoFe/NFF; (b) TOF values at the overpotential of 300 mV of the corresponding samples; Figure S16: Specific activity of LaCoFe/NFF, La0.9CoFe/NFF and La0.8CoFe/NFF recorded at an overpotential of 270 mV; Figure S17: Chronopotentiometry test for 20 h at 10 mA cm^−2^ of electrocatalysts; Figure S18. SEM images of La0.9CoFe/NFF after OER long-term tests in 1 M KOH; Figure S19: XPS spectra of (a) La 3d, (b) Co 2p (c) Fe 2p and (d) O 1s of La0.9CoFe/NFF after long-term OER tests in 1M KOH; Table S1: XPS Peak Positions (Binding Energy, eV) Obtained for All the LaxCoFe/NFF catalysts; Table S2: Comparison of HER performance of La0.9CoFe/NFF with other perovskite electrocatalysts reported previously; Table S3: Comparison of OER performance of La0.9CoFe/NFF with other perovskite electrocatalysts reported previously; Table S4: Comparison of Cell voltage of La0.9CoFe/NFF||La0.9CoFe/NFF with perovskite electrocatalysts reported previously. References are cited from [46,47,48,49,50,51,52,53,54,55,56,57].
