# Supplementary material for: Controllable La Deficiency Engineering within Perovskite Oxides for Enhanced Overall Water Splitting
Source: Molecules. 2024 Mar 18;29(6):1342. doi: 10.3390/molecules29061342 (PMC10975698; doi:10.3390/molecules29061342)
Supplement: Supplementary file 1 [file molecules-29-01342-s001.zip › molecules-2922639-supplementary.pdf]

Supporting Information

Controllable La deficiency engineering within perovskite oxides for enhanced overall water splitting

Xiaohu Xu [1*†], Kaiwei Guo [1†], Xinyue Yu [1*]

[1] College of Physics and Information Engineering, Shanxi Normal University, No.339 Taiyu road, Xiaodian District, Taiyuan 030031, China; bigbrowm@163.com (X.H.X), ewe32e3@163.com (K.W.G), yuxinyue9907@163.com (X.Y.Y);

[†] These authors contributed equally to this work.

* Author to whom correspondence should be addressed; bigbrowm@163.com (X.H.X); yuxinyue9907@163.com (X.Y.Y).

Number of pages: 25

Number of figures: 19

Number of tables: 4

Content

Overall XPS survey spectra .... **Figure S1**

LSV curves of various catalysts with different hydrothermal temperature. ... **Figure S2**

LSV curves of various catalysts with different Co/Fe ratio .... **Figure S3**

LSV curves of LaCoFe/NF and LaCoFe/NFF. .... **Figure S4**

Cyclic voltammograms with different scanning rate for HER .... **Figure S5**

Electrochemical active area (ECSA) of HER .... **Figure S6**

Reduction peaks recorded at 0.2 V s^{-1}.. .... **Figure S7**

TOF curves and TOF values at the overpotential of 200 mV .... **Figure S8**

BET surface area calculations and specific activity .... **Figure S9**

Chronopotentiometry test for HER .... **Figure S10**

SEM images after long-term HER tests .... **Figure S11**

XPS spectra after long-term HER tests .... **Figure S12**

Cyclic voltammograms with different scanning rate for OER .... **Figure S13**

Electrochemical active area (ECSA) of OER .... **Figure S14**

TOF curves and TOF values at the overpotential of 300 mV .... **Figure S15**

Specific activity. .... **Figure S16**

Chronopotentiometry test for OER .... **Figure S17**

SEM images after long-term OER tests. .... **Figure S18**

XPS spectra after long-term OER tests. .... **Figure S19**

XPS Peak Positions. .... **Table S1**

Comparison of HER performance and Tafel .... **Table S2**

Comparison of OER performance. .... **Table S3**

Comparison of Cell voltage .... **Table S4**

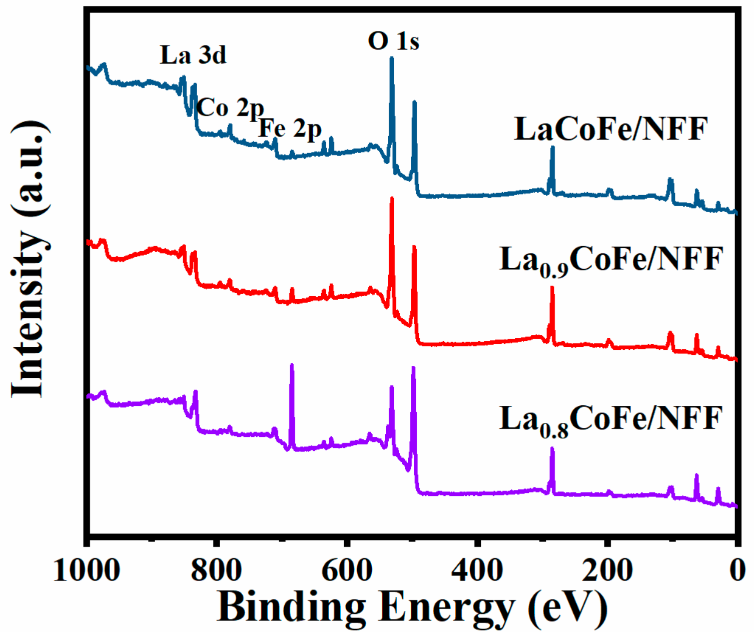

Figure S1. Overall XPS survey spectra of LaCoFe/NFF, $La_{0.9}CoFe/NFF$ and $La_{0.8}CoFe/NFF$.

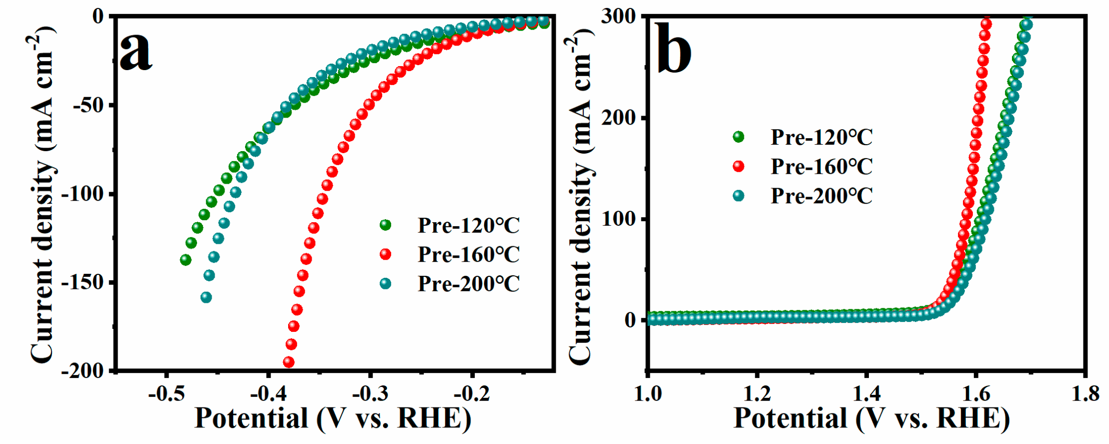

Figure S2. LSV curves of LaCoFe/NFF catalysts with different hydrothermal temperature in 1 M KOH solution for (a) HER and (b) OER.

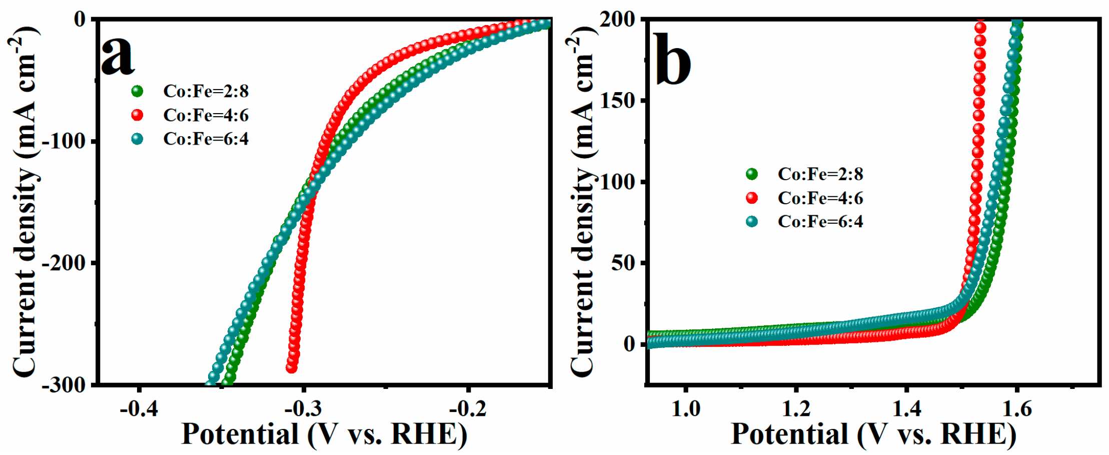

Figure S3. LSV curves of various catalysts with different Co/Fe ratio in 1 M KOH solution for (a) HER and (b) OER.

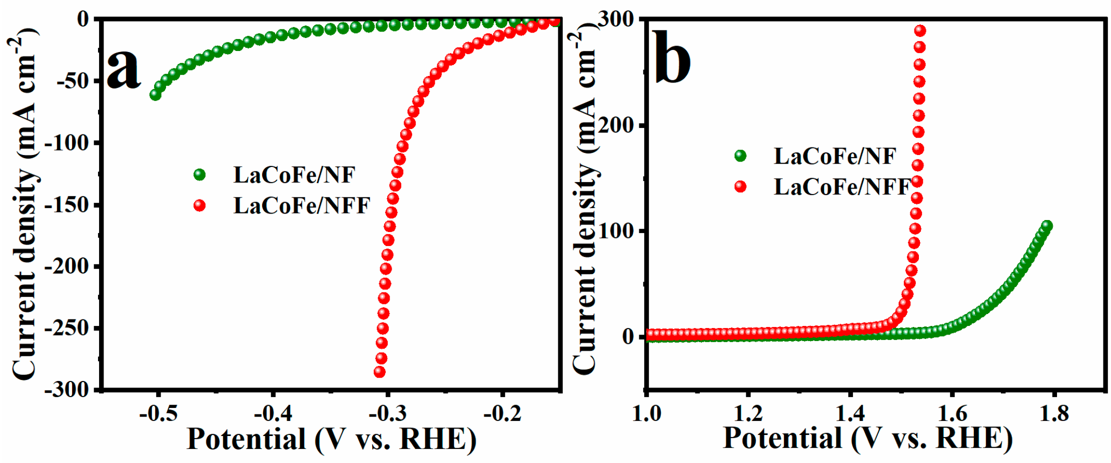

Figure S4. LSV curves of LaCoFe/NF and LaCoFe/NFF in 1 M KOH solution for (a) HER and (b) OER.

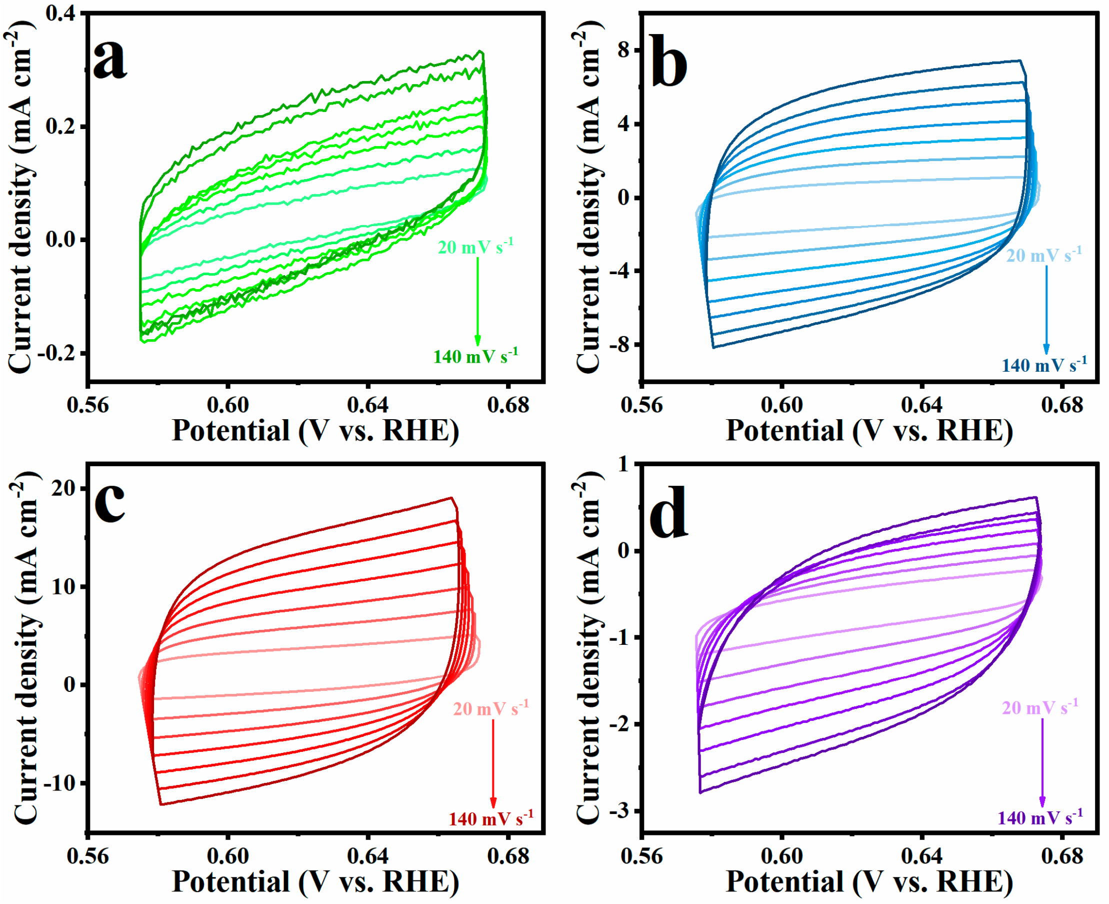

Figure S5. Cyclic voltammetry (CVs) curves for HER in a non-faradic current region (potential window at 0.57 -0.67 V (vs. RHE)) at different scan rates (20, 40, 60, 80, 100, 120 and 140 mV s^{-1}) of (a) NFF, (b) LaCoFe/NFF, (c) $La_{0.9}$CoFe/NFF and (d) $La_{0.8}$CoFe/NFF.

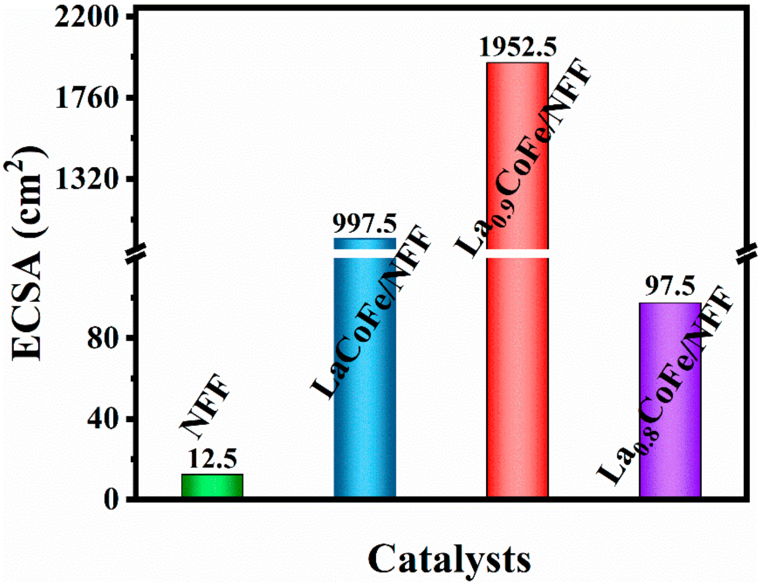

Figure S6. Electrochemical active area (ECSA) of HER.

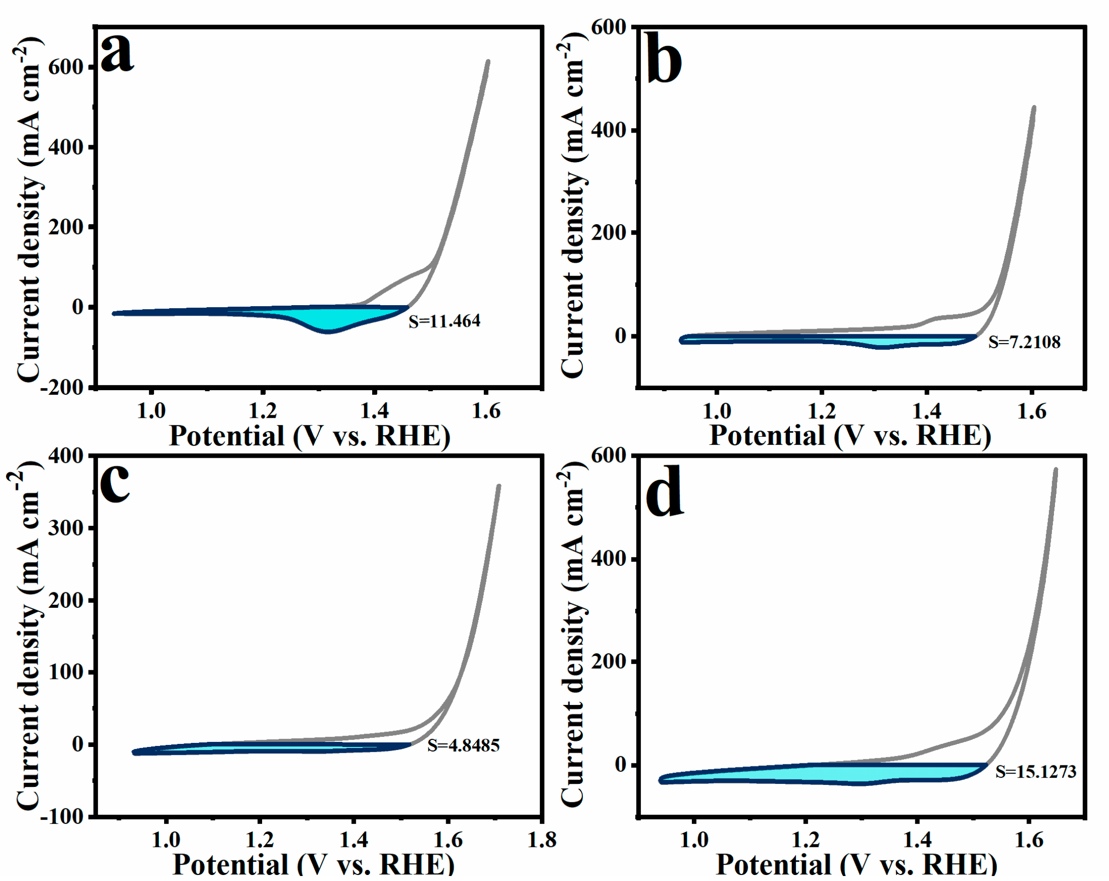

Figure S7. Reduction peaks recorded at 0.2 V s^{-1}. (a) NFF; (b) LaCoFe/NFF; (c) $La_{0.9}$CoFe/NFF; (d) $La_{0.8}$CoFe/NFF.

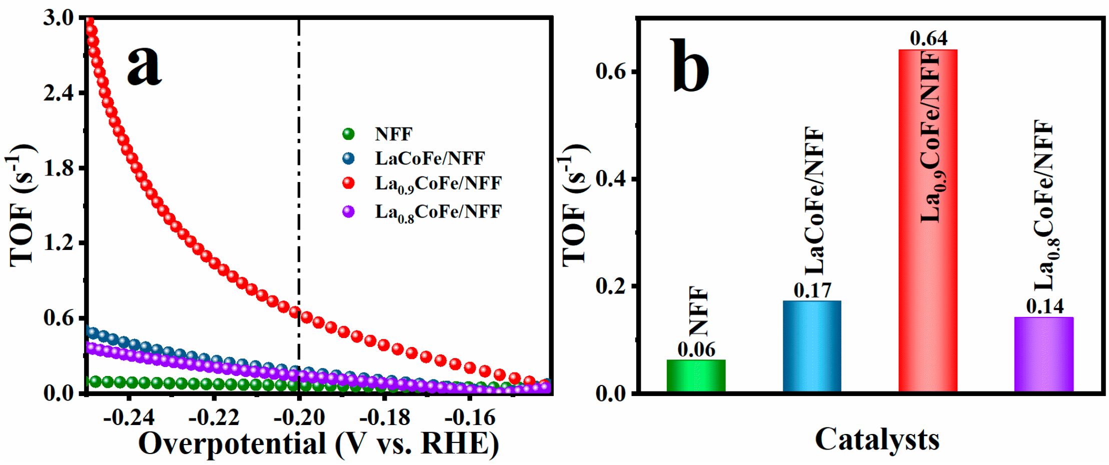

Figure S8. (a) The potential dependent TOF curves of the NFF, LaCoFe/NFF, $La_{0.9}CoFe/NFF$ and $La_{0.8}CoFe/NFF$; (b) TOF values at the overpotential of 200 mV of the corresponding samples.

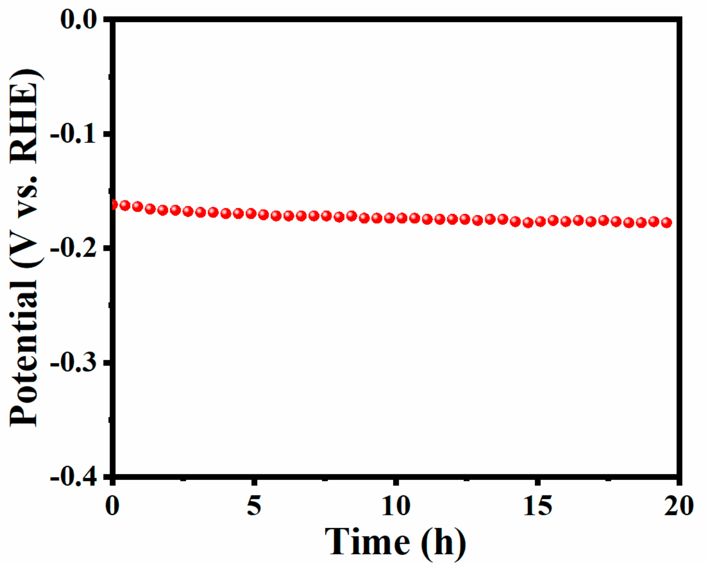

Figure S9. Chronopotentiometry test for 20 h at -10 mA cm^{-2} of electrocatalysts.

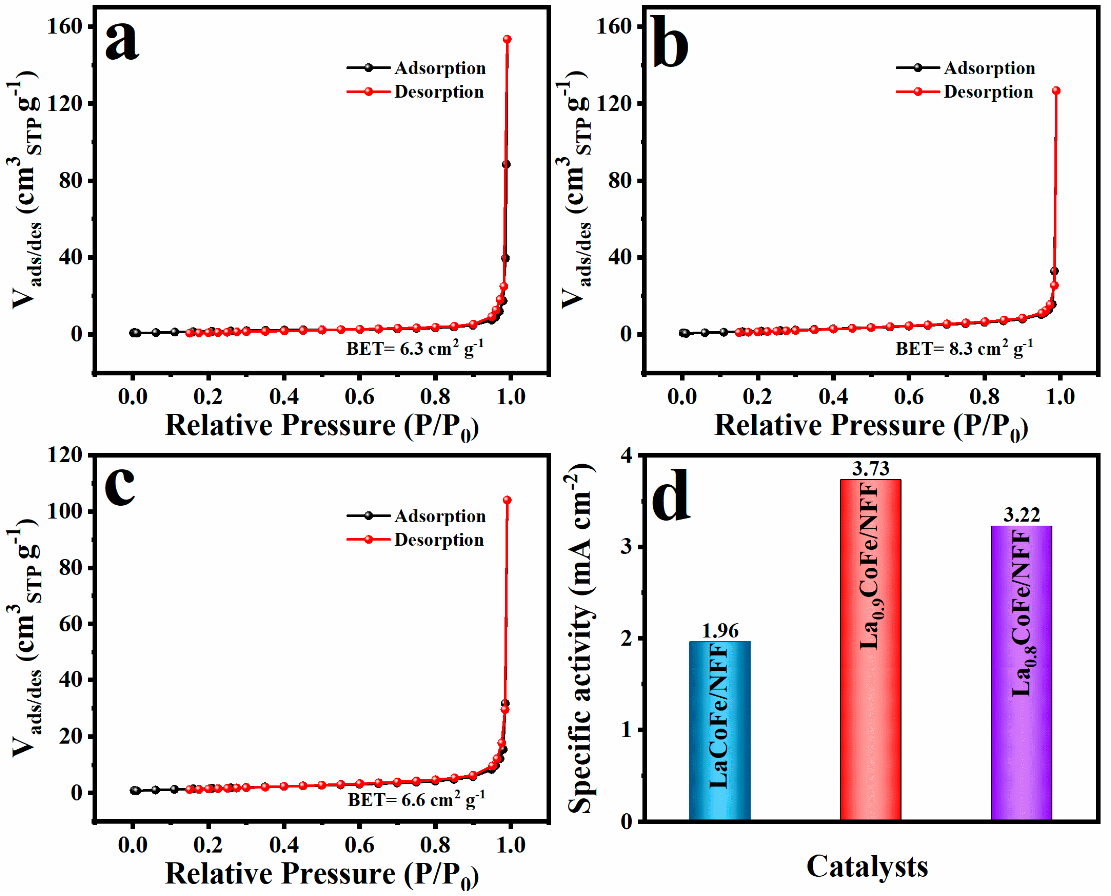

Figure S10. BET surface area calculations from the N_2 absorption-desorption isotherms. (a) LaCoFe/NFF, (b) $La_{0.9}CoFe/NFF$ and (c) $La_{0.8}CoFe/NFF$; (d) Specific activity of LaCoFe/NFF, $La_{0.9}CoFe/NFF$ and $La_{0.8}CoFe/NFF$ recorded at an overpotential of 200 mV.

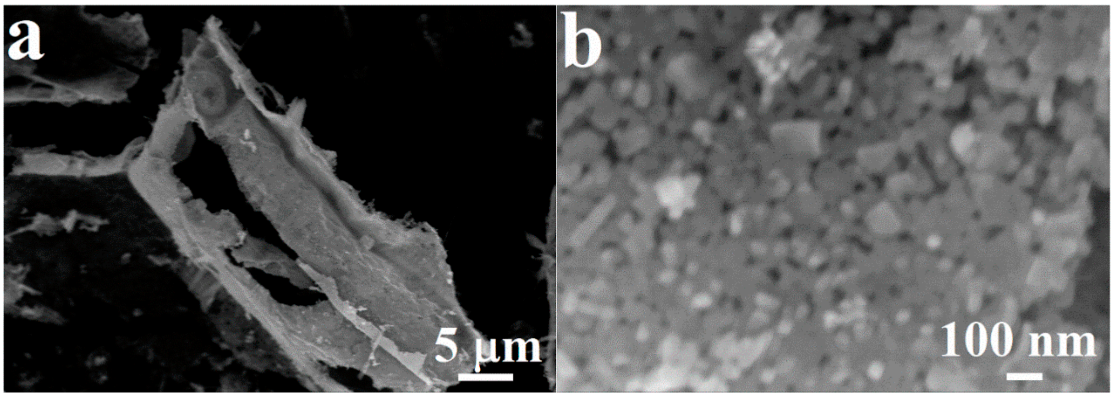

Figure S11. SEM images of $La_{0.9}CoFe/NFF$ after HER long-term tests in 1 M KOH.

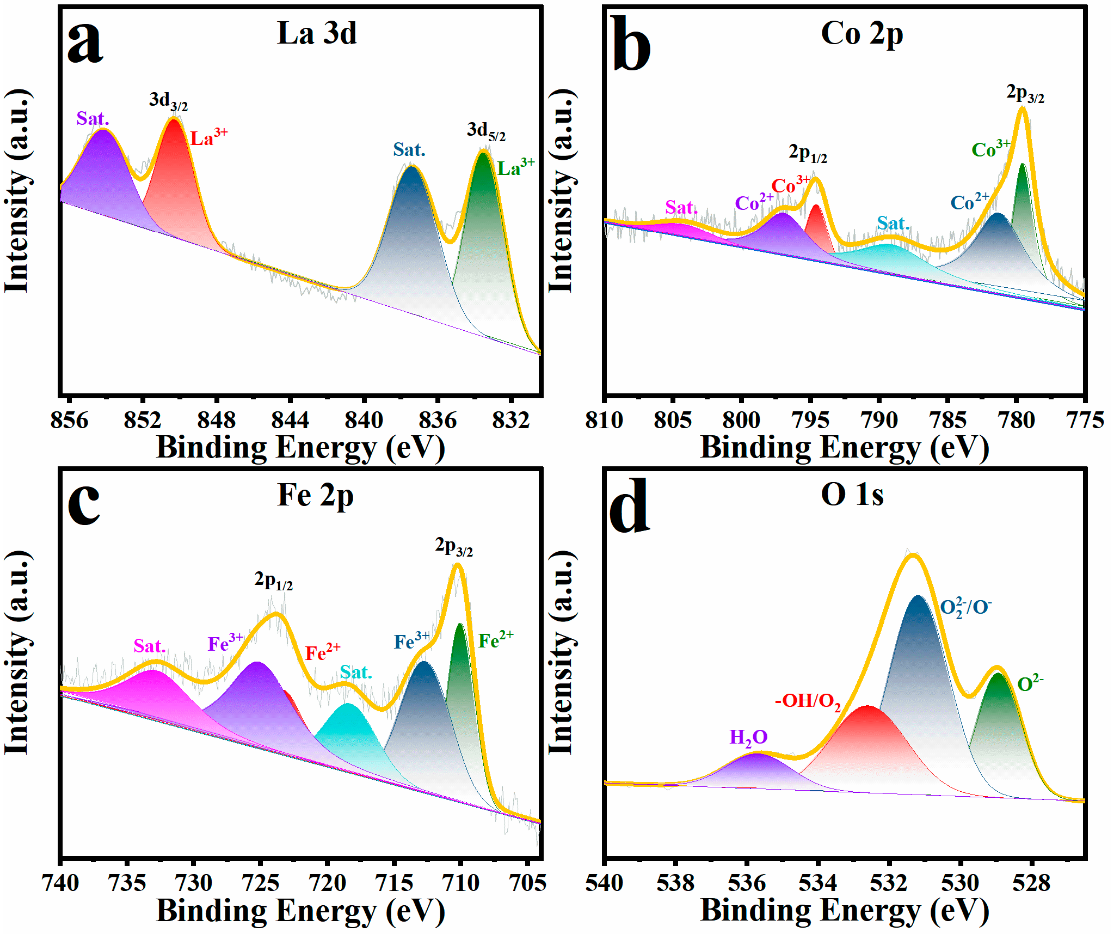

Figure S12. XPS spectra of (a) La 3d, (b) Co 2p (c) Fe 2p and (d) O 1s of $La_{0.9}CoFe/NFF$ after HER tests in 1M KOH.

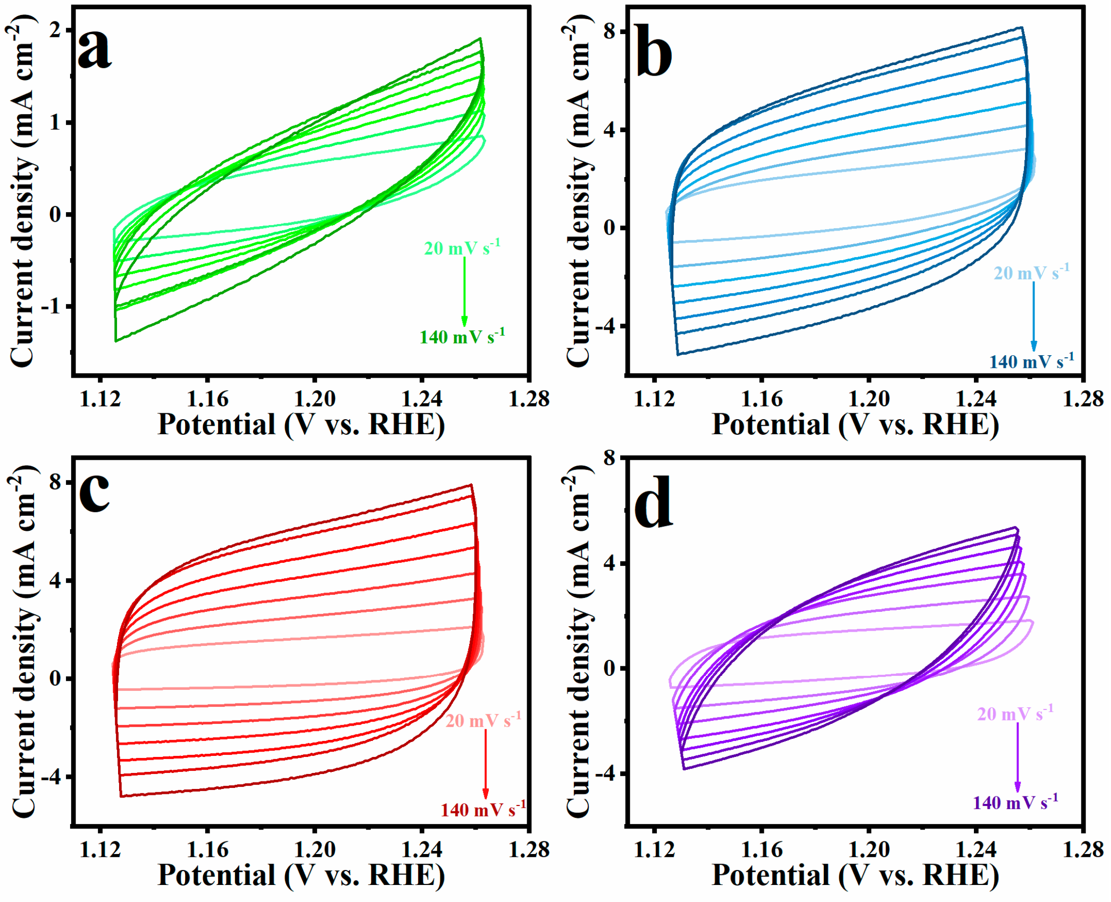

Figure S13. Cyclic voltammetry (CVs) curves for OER in a non-faradic current region (potential window at 1.12 - 1.26 V (vs. RHE)) at different scan rates (20, 40, 60, 80, 100, 120 and 140 mV s^{-1}) of (a) NFF, (b) LaCoFe/NFF, (c) $La_{0.9}$CoFe/NFF and (d) $La_{0.8}$CoFe/NFF.

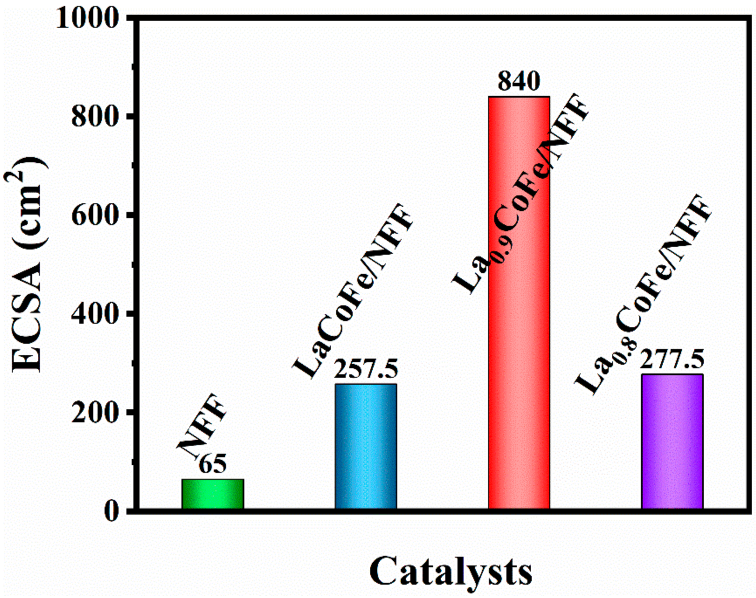

Figure S14. Electrochemical active area (ECSA) of OER.

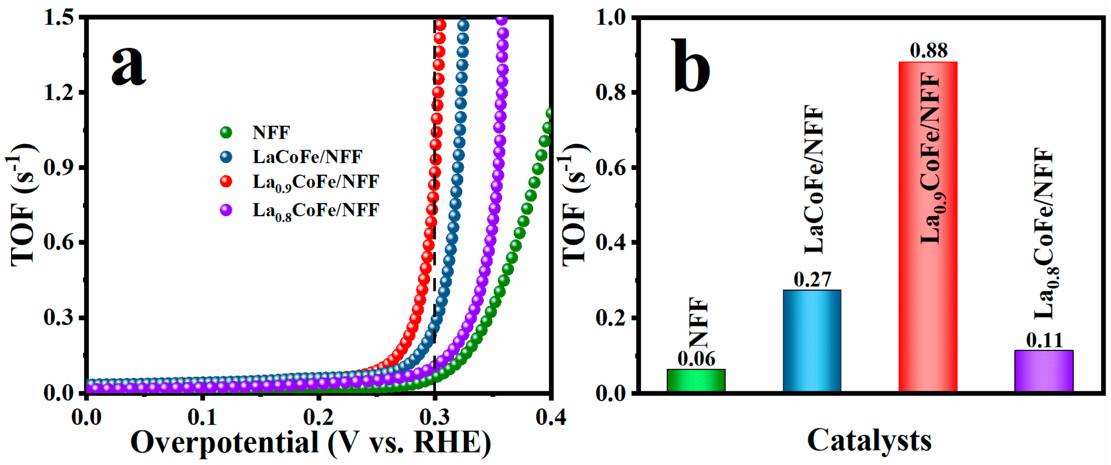

Figure S15. (a) The potential dependent TOF curves of the NFF, LaCoFe/NFF, $La_{0.9}CoFe/NFF$ and $La_{0.8}CoFe/NFF$; (b) TOF values at the overpotential of 300 mV of the corresponding samples.

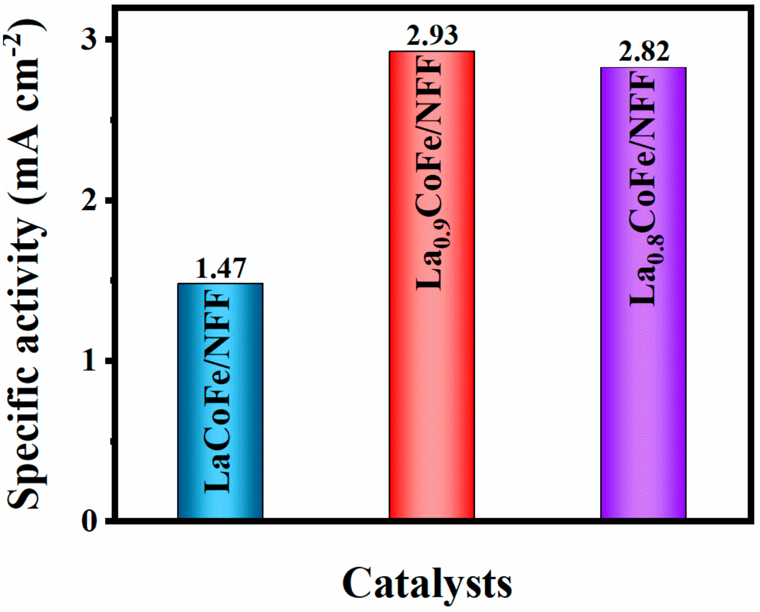

Figure S16. Specific activity of LaCoFe/NFF, $La_{0.9}$CoFe/NFF and $La_{0.8}$CoFe/NFF recorded at an overpotential of 270 mV.

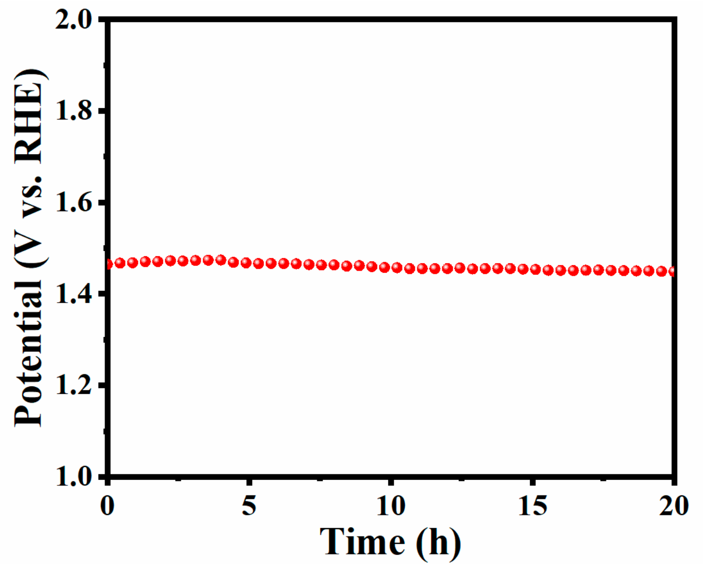

Figure S17. Chronopotentiometry test for 20 h at 10 mA cm^{-2} of electrocatalysts.

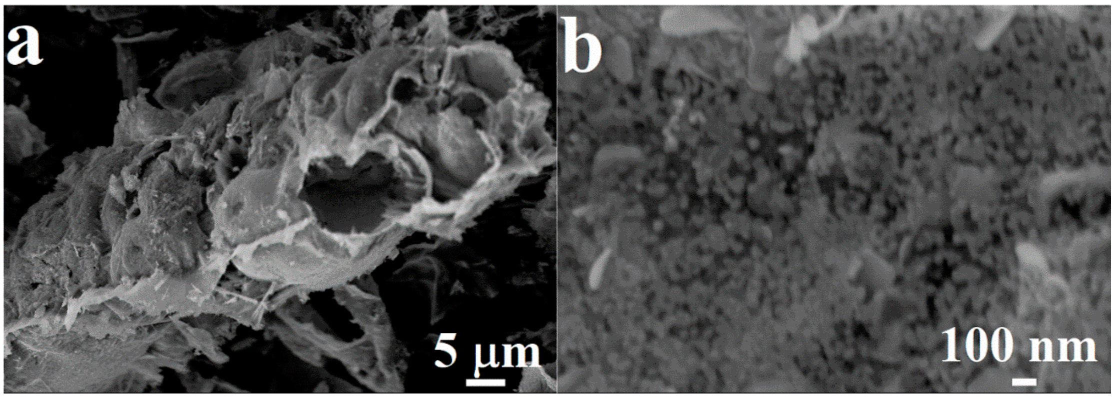

Figure S18. SEM images of $La_{0.9}CoFe/NFF$ after OER long-term tests in 1 M KOH.

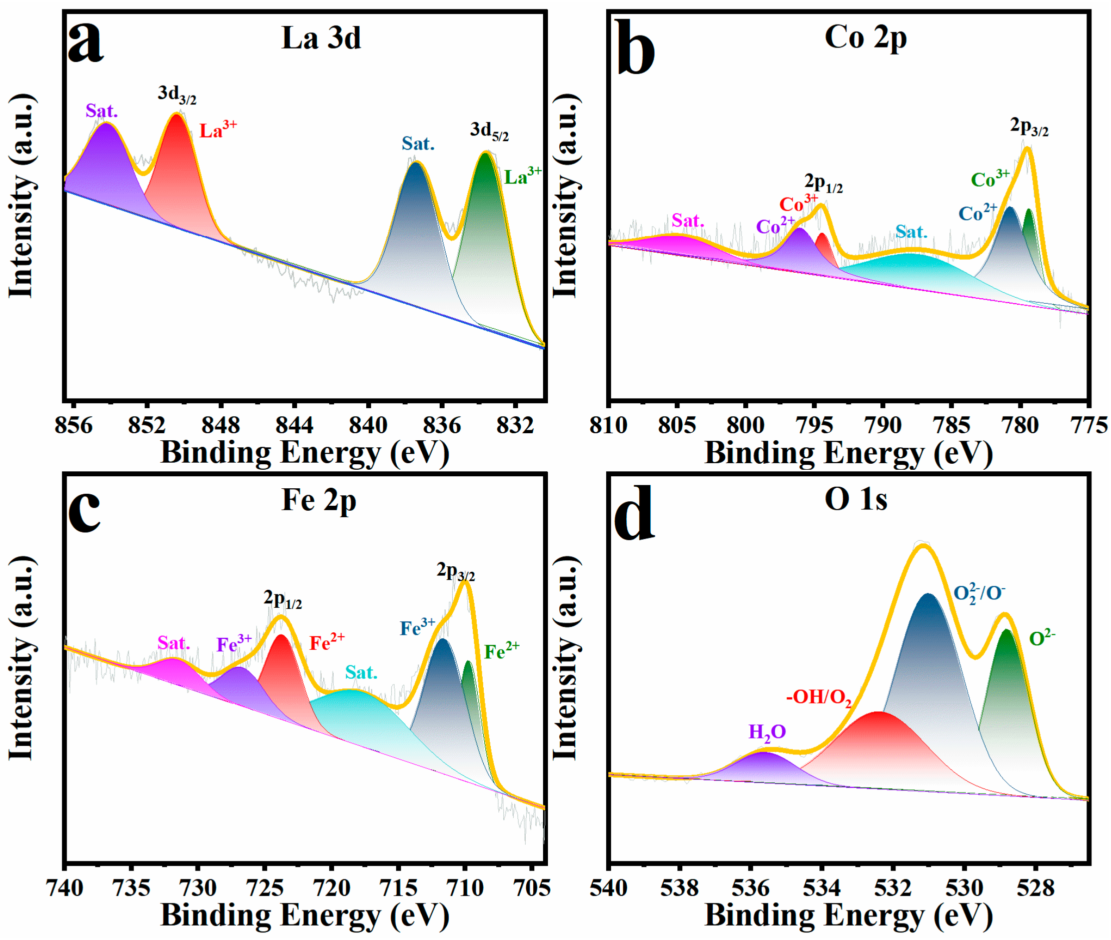

Figure S19. XPS spectra of (a) La 3d, (b) Co 2p (c) Fe 2p and (d) O 1s of $La_{0.9}$CoFe/NFF after long-term OER tests in 1M KOH.

Table S1. XPS Peak Positions (Binding Energy, eV) Obtained for All the La_xCoFe/NFF catalysts.

XPS peaks	LaCoFe/NFF	$La_{0.9}CoFe/NFF$	$La_{0.8}CoFe/NFF$
**La $3d_{5/2}$**	833.4	833.4	832.6
	837.1	837.3	837.1
**La $3d_{3/2}$**	850.2	850.2	850.1
	854.0	854.0	854.1
**Co $2p_{3/2}$**	779.3	778.5	779.5
	780.6	781.2	781.8
	786.9	790.0	788.9
**Co $2p_{1/2}$**	784.4	794.5	794.4
	796.0	796.8	796.7
	804.5	804.1	802.2
**Fe $2p_{3/2}$**	790.4	709.9	709.4
	711.5	712.6	711.8
	717.6	718.2	717.7
**Fe $2p_{1/2}$**	723.6	723.2	723.5
	726.6	725.0	725.6
	731.4	732.6	732.1
**O 1s**	528.8	528.9	528.8
	531.0	531.1	831.1
	532.3	532.5	532.3
	535.6	535.7	536.8

Table S2. Comparison of HER performance of $La_{0.9}$CoFe/NFF with other perovskite electrocatalysts reported previously.

Catalyst	Electrolyte	Tafel slope ($mVdec^{-1}$)	η(mV) @ j=10 $mA\ cm^{-2}$	η(mV) @ j=100 $mA\ cm^{-2}$	Reference
**$La_{0.9}$CoFe/NFF**	**1 M KOH**	**80.8**	**160.5**	**241**	**This work**
LCC4	1 M NaOH	144	305	NA	1
CoP-PBSCF	1 M KOH	93.8	240	NA	2
C-NSCFNb	1 M KOH	133	470	NA	4
$Ca_2FeCoO_{6-\delta}$	1 M KOH	105	250	NA	17
LSFCP-55	1 M KOH	119.2	280	560	45
$La_{0.7}Y_{0.3}Co_{0.5}Ni_{0.5}O_3$	1 M KOH	156	400	NA	46
$BaNiO_3$	1 M KOH	427	216	~670	47
$BaSrCoMoO_6$	1 M KOH	160	325	NA	48
S9	1 M KOH	212	451	~700	49
LCO(110)	1 M KOH	96.1	390	NA	50
LCO/MoS_2-4	1 M KOH	78	241	NA	51
STFP-1	2 M KOH	77	182.4	NA	52
STFN/CNT-700	0.1 M KOH	116	320	NA	53
AC-$SrIrO_3$	0.1 M KOH	49	370	NA	54
$Co(OH)_2$/SFM-NF	0.1 M KOH	77	312	NA	55
r-LSCN-P	0.1 M KOH	105	339	NA	56

NA: Not applicable since not reported.

Table S3. Comparison of OER performance of $La_{0.9}CoFe/NFF$ with other perovskite electrocatalysts reported previously.

Catalyst	Electrolyte	Tafel slope ($mVdec^{-1}$)	η(mV) @ j=10 mA cm^{-2}	η(mV) @ j=100 mA cm^{-2}	Reference
**$La_{0.9}CoFe/NFF$**	**1 M KOH**	**59.1**	**234.7**	**296.1**	**This work**
LCC4	1 M NaOH	80	380	NA	1
CoP-PBSCF	1 M KOH	81.5	340	NA	2
C-NSCFNb	1 M KOH	89	420	NA	4
$Ca_2FeCoO_{6-\delta}$	1 M KOH	57	250	NA	17
LSFCP-55	1 M KOH	53.2	330	420	45
$La_{0.7}Y_{0.3}Co_{0.5}Ni_{0.5}O_3$	1 M KOH	188	338	NA	46
BU	1 M KOH	64	253	400	47
$BaSrCoMoO_6$	1 M KOH	81	400	NA	48
SU	1 M KOH	70	259	410	49
LSCO(111)	1 M KOH	86.9	289	NA	50
LCO/MoS_2-4	1 M KOH	62.5	370	NA	51
STFP-1	2 M KOH	73	277.6	420	52
STFN/CNT-700	0.1 M KOH	98	480	NA	53
$SrIrO_3$	0.1 M KOH	55	300	NA	54
$Co(OH)_2$/SFM-NF	0.1 M KOH	71	387	NA	55
r-LSCN-P	0.1 M KOH	72.1	410	NA	56
S-LCF	1 M KOH	60	360	NA	57

NA: Not applicable since not reported.

Table S4. Comparison of Cell voltage of $La_{0.9}CoFe/NFF||La_{0.9}CoFe/NFF$ with perovskite electrocatalysts reported previously.

Catalyst	Electrolyte	Cell Voltage (V) @ j=10 mA cm^{-2}	Reference
**$La_{0.9}CoFe/NFF||La_{0.9}CoFe/NFF$**	**1 M KOH**	**1.573**	**This work**
CoP-PBSCF||CoP-PBSCF	1 M KOH	1.69	2
C-NSCFNb||C-NSCFNb	1 M KOH	1.71	4
LSFCP-55||LSFCP-55	1 M KOH	1.57	45
$La_{0.7}Y_{0.3}Co_{0.5}Ni_{0.5}O_3||La_{0.7}Y_{0.3}Co_{0.5}Ni_{0.5}O_3$	1 M KOH	1.63	46
BU||BU	1 M KOH	1.82	47
SU||S9	1 M KOH	1.88	49
STFP01||STFP01	2 M KOH	1.665	52
STFN/CNT-700||STFN/CNT-700	0.1 M KOH	1.8	53
AC-$SrIrO_3||SrIrO_3$	0.1 M KOH	1.59	54
$Co(OH)_2$||SMF-NF	0.1 M KOH	1.6	55
r-LSCN-P||r-LSCN-P	0.1 M KOH	1.7	56
S-LCF||S-LCF	1 M KOH	1.67	57

References

46. Hui, J.; Yan, C.; Shi, Y.; Yang, Z. Rare-earth metal multicomponent perovskite as a electrocatalyst for water splitting. *Mater. Lett.* **2023**, *332*, 133532. https://doi.org/10.1016/j.matlet.2022.133532.
47. Junita, J.; Jayalakshmi, D.; Rodney, J.D. Combustion-derived $BaNiO_3$ nanoparticles as a potential bifunctional electrocatalyst for overall water splitting. *Int. J. Hydrogen Energy* **2023**, *48*, 14287–14298. https://doi.org/10.1016/j.ijhydene.2022.12.291.
48. Karki, S.B.; Andriotis, A.N.; Menon, M.; Ramezanipour, F. Enhancement of Electrocatalytic Activity for both Hydrogen and Oxygen Evolution Reactions of a Perovskite Oxide. *J. Phys. Chem. C* **2022**, *126*, 20011–20019. https://doi.org/10.1021/acs.jpcc.2c05651.
49. Junita, J.; Jayalakshmi, D.; Rodney, J.D. Effect of annealing temperature on the bifunctional electrocatalytic properties of strontium nickelate ($SrNiO_3$) nanoparticles for efficient overall water splitting. *Int. J. Hydrogen Energy* **2022**, *47*, 30602–30612. https://doi.org/10.1016/j.ijhydene.2022.07.007.
50. Zhou, Y.-N.; Wang, F.-G.; Zhen, Y.-N.; Nan, J.; Dong, B.; Chai, Y.-M. Crystal facet engineering of perovskite cobaltite with optimized electronic regulation for water splitting. *Sci. China Mater.* **2022**, *65*, 2665–2674. https://doi.org/10.1007/s40843-022-2016-5.
51. Rana, A.K.; Jeong, M.H.; Noh, Y.I.; Park, H.; Baik, J.M.; Choi, K.J. Phase-Tuned MoS_2 and Its Hybridization with Perovskite Oxide as Bifunctional Catalyst: A Rationale for Highly Stable and Efficient Water Splitting. *ACS Appl. Mater. Interfaces* **2022**, *14*, 18248–18260. https://doi.org/10.1021/acsami.1c21425.
52. Sarmad, Q.; Khan, U.M.; Baig, M.M.; Hassan, M.; Butt, F.A.; Khoja, A.H.; Liaquat, R.; Khan, Z.S.; Anwar, M.; Muhammed Ali, S.A. Praseodymium-doped $Sr_2TiFeO_{6-\delta}$ double perovskite as a bi-

functional electrocatalyst for hydrogen production through water splitting. *J. Environ. Chem. Eng.* **2022**, *10*, 107609. https://doi.org/10.1016/j.jece.2022.107609.
53. Wu, X.; Yu, J.; Yang, G.; Liu, H.; Zhou, W.; Shao, Z. Perovskite oxide/carbon nanotube hybrid bifunctional electrocatalysts for overall water splitting. *Electrochim. Acta* **2018**, *286*, 47–54. https://doi.org/10.1016/j.electacta.2018.08.010.
54. Yu, J.; Wu, X.; Guan, D.; Hu, Z.; Weng, S.-C.; Sun, H.; Song, Y.; Ran, R.; Zhou, W.; Ni, M.; et al. Monoclinic $SrIrO_3$: An Easily Synthesized Conductive Perovskite Oxide with Outstanding Performance for Overall Water Splitting in Alkaline Solution. *Chem. Mater.* **2020**, *32*, 4509–4517. https://doi.org/10.1021/acs.chemmater.0c00149.
55. He, B.; Tan, K.; Gong, Y.; Wang, R.; Wang, H.; Zhao, L. Coupling amorphous cobalt hydroxide nanoflakes on $Sr_2Fe_{1.5}Mo_{0.5}O_{5+\delta}$ perovskite nanofibers to induce bifunctionality for water splitting. *Nanoscale* **2020**, *12*, 9048–9057. https://doi.org/10.1039/d0nr00848f.
56. Wang, Y.; Wang, Z.; Jin, C.; Li, C.; Li, X.; Li, Y.; Yang, R.; Liu, M. Enhanced overall water electrolysis on a bifunctional perovskite oxide through interfacial engineering. *Electrochim. Acta* **2019**, *318*, 120–129. https://doi.org/10.1016/j.electacta.2019.06.073.
57. Tang, L.; Chen, Z.; Zuo, F.; Hua, B.; Zhou, H.; Li, M.; Li, J.; Sun, Y. Enhancing perovskite electrocatalysis through synergistic functionalization of B-site cation for efficient water splitting. *Chem. Eng. J.* **2020**, *401*, 126082. https://doi.org/10.1016/j.cej.2020.126082.
